# Predictive Value of Geriatric Nutritional Risk Index in Patients With Lower Extremity Peripheral Artery Disease: A Meta-Analysis

**DOI:** 10.3389/fnut.2022.903293

**Published:** 2022-06-22

**Authors:** Guodong Liu, Chen Zou, Yu Jie, Pei Wang, Xiaoyan Wang, Yu Fan

**Affiliations:** ^1^Department of General Surgery, The Suqian Clinical College of Xuzhou Medical University, Suqian, China; ^2^Cancer Institute, The Affiliated People's Hospital, Jiangsu University, Zhenjiang, China; ^3^Department of Gastroenterology, The Suqian Clinical College of Xuzhou Medical University, Suqian, China

**Keywords:** geriatric nutritional risk index, peripheral artery disease, major adverse cardiovascular and leg events, all-cause mortality, amputation, meta-analysis

## Abstract

**Background:**

Conflicting results have been reported on the value of the Geriatric Nutritional Risk Index (GNRI) in predicting adverse outcomes in patients with peripheral artery disease (PAD). The objective of this meta-analysis was to evaluate the association of GNRI with adverse outcomes in patients with lower extremity PAD.

**Methods:**

Relevant studies were comprehensively searched in PubMed and Embase databases until December 31, 2021. Eligible studies should evaluate the value of GNRI in predicting major adverse cardiovascular and leg events (MACLEs), all-cause mortality, and amputation in patients with lower extremity PAD.

**Results:**

Eight studies reporting on 9 articles involving 5,541 patients were included. A fixed-effect model meta-analysis showed that patients with PAD with low GNRI had an increased risk of MACLEs [adjusted risk ratio (RR) 2.26; 95% confidence interval (CI) 1.54–3.31] and all-cause mortality (RR 2.38; 95% CI 1.71–3.31) compared with those with high GNRI. When analysis of GNRI is by continuous data, 10 units of GNRI decrease was associated with 36% and 44% higher risk of MACLEs and all-cause mortality, respectively. However, per 10 units GNRI score decrease was not significantly associated with a higher risk of amputation (*p* = 0.051).

**Conclusion:**

Low GNRI may be an independent predictor of adverse outcomes in patients with lower extremity PAD. Routine screening of nutritional status using the GNRI may provide important prognostic information in patients with PAD.

## Introduction

Peripheral artery disease (PAD) refers to a common condition of narrowing or blocking arteries outside the heart, affecting over 200 million people worldwide (1). The weighted mean age-standardized prevalence of outpatient PAD was 11.8% (2). Despite progress in evidence-based management, PAD is still associated with a substantially higher risk of mortality (3) and limb loss (4). Therefore, early risk stratification remains very important for improving the personalized management of PAD.

Malnutrition is associated with poor survival in various populations including hospitalized patients with chronic limb-threatening ischemia (CLTI) (5). The reported prevalence of malnutrition was between 22 and 75% in patients with PAD (5, 6). Geriatric Nutritional Risk Index (GNRI) is a simple scoring system for the assessment of nutritional status in the aging population. This formula is calculated as follows: GNRI = 1.489 × serum albumin (g/l) + 41.7 × present body weight/ideal body weight (7). Under this nutritional tool, a low GNRI score reflects poor nutritional status (8). The GNRI scores of 92–98, 82–91, and <82 reflect mild, moderate, and severe malnutrition. Individuals with a GNRI score of 92 or below are grouped as malnutrition (mild 92–98, moderate 82–91, and severe <82). Accumulating evidence suggested that low GNRI scores may be linked with adverse outcomes in patients with lower extremity PAD (9–13). However, conflicting findings existed on the predictive value of GNRI score in these patients (14–16).

No previous meta-analysis has systematically evaluated the predictive value of the GNRI score in lower extremity patients with PAD. The current meta-analysis aimed to evaluate the value of the low GNRI score in predicting adverse outcomes in patients with lower extremity PAD in terms of major adverse cardiovascular and leg events (MACLEs), all-cause mortality, and amputation.

## Methods

### Literature Search

The report of this study followed the guidelines of Preferred Reporting Items for Systematic Reviews and Meta-Analyses (17). Two independent authors systematically searched PubMed and Embase databases from their inceptions to December 31, 2021. A combination of the following keywords was applied as a search strategy: “Geriatric Nutritional Risk Index (Mesh term)” AND “peripheral arterial disease (Mesh term)” OR “peripheral artery disease (Mesh term)” OR “lower extremity arterial diseases (Free term)” OR “critical limb ischemia (Free term)” OR “chronic limb-threatening ischemia (Free term)” OR “intermittent claudication (Free term).” Reference lists of pertinent articles were also manually scanned for additional studies. No language restriction was input for the literature search. Our meta-analysis was not prospectively registered in the PROSPERO database.

### Inclusion and Exclusion Criteria

Studies satisfying all the following criteria were included: (1) population: patients with a diagnosis of lower extremity PAD; (2) exposure: GNRI score at baseline; (3) comparison: patients with low GNRI score *vs*. those with high GNRI score; (4) outcome measures: MACLEs, all-cause mortality, and amputation; (5) study design: retrospective or prospective observational studies; and (6) provided multivariable-adjusted risk estimate for outcomes of interest according to categorical or continuous GNRI score. MACLEs were defined as total death, limb surgery (amputation, target vessel revascularization, or endovascular therapy), stroke, myocardial infarction, and admission for CLTI or cardiovascular disease. For articles enrolling patients from the same population, we selected the articles with the longest follow-up. The exclusion criteria included: (1) studies did not provide a detailed risk estimate; (2) nutritional status determined by other scoring tools; (3) outcome measures were not of interest, and (4) follow-up duration <3 months.

### Data Extraction and Quality Assessment

Data extraction and quality assessment were performed by two independent authors. Any discrepancy was settled by consensus. The data extracted included: the author's surname, publication year, the origin of study, study design, sample sizes, percentage of men, age at baseline, assessment of MACLEs, cutoff of low GNRI score, outcome measures, length of follow-up, fully adjusted risk estimate, and degree variables in the adjustment. Assessment of the methodological quality of the included studies was performed using a 9-point Newcastle-Ottawa Scale (NOS) (18). Studies with a score of 7 points or over were considered high-quality.

### Data Synthesis and Analysis

Data synthesis and analysis were conducted using Stata 12.0 (Stata Corporation, College Station, TX). The predictive value of the GNRI score was calculated by pooling a multivariable-adjusted risk ratio (RR) with a 95% confidence interval (CI) for low *vs*. high GNRI group or per 10 units GNRI decrease. When analyzing the predictive value of GNRI score by continuous data, we recalculated RR per 10 units GNRI decrease by the following formula: RR10 = exp (ln (RR1) ×10). Cochrane *Q* test and I^2^ statistic were applied to investigate the heterogeneity, with statistical significance set at *p* < 0.10 or I^2^ ≥50%. A fixed-effect model was selected in cases without evidence of significant heterogeneity. Sensitivity analysis was conducted using the leave-out one-study method. Publication bias was scheduled using the Begg's test (19) and Egger's test (20) when at least 10 studies were included in the analyzed outcomes. Subgroup analysis was performed according to study design, sample sizes, region, types of patients, median/mean age, and duration of follow-up.

## Results

### Search Results and Study Characteristics

**Figure 1:**
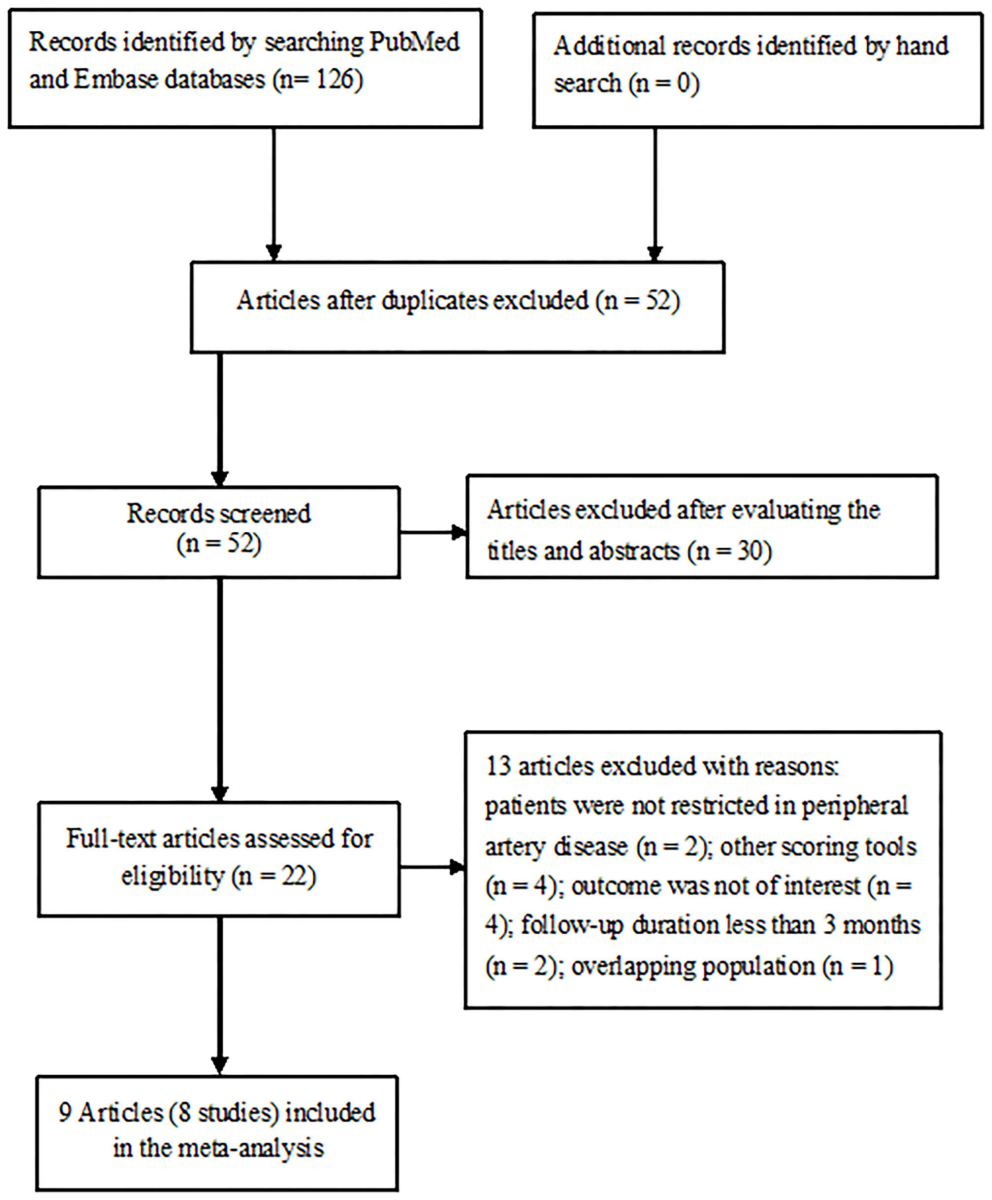
Flow chart showing studies selection process.

The initial literature yielded a total of 126 potentially relevant records. A total of 58 articles were left after the removal of duplications. Of which, 30 articles were excluded after reviewing the titles or abstracts and 22 full-text articles were retrieved for detailed evaluation. Thirteen articles were further excluded after applying our inclusion and exclusion criteria. Two articles (12, 14) from the same population analyzed the outcomes by categorical and continuous GNRI scores. Thus, 8 studies reporting on 9 articles (9, 11–16, 21, 22) were finally included in this meta-analysis (Figure 1).

Details of the study characteristics are described in Table 1. These studies were published from 2016 to 2021 and performed in Japan (11–15, 21, 22), China (9), Taiwan (13), and the USA (16). Four studies (11, 13, 15, 21) were prospective designs and others were retrospective studies. Sample sizes ranged between 172 and 2,246, with a total of 5,541 patients. The duration of follow-up ranged between 1.2 and 6.1 years. Based on the NOS criteria, all included studies were classified as having high methodological quality (Table 2).

**Table 1:** Main characteristics of the included studies.

**Author/year**	**Region**	**Study design**	**Patients (% male)**	**Age (years)**	**Therapy**	**Follow-up (years)**	**Analysis of GNRI**	**Definition of MACLEs**	**Outcomes/HR (95% CI)**	**Adjusted for variables**
Luo et al. (9)	China	R	CLTI 172 (53.6)	72.0 ± 3.1	Multiple therapies	3.0	Per 10 units decrease	—	Amputation 1.63 (1.10–2.37)	Age, albumin, BMI, DM, LDL, TC
Yokoyama et al. (11)	Japan	P	PAD 357 (80.4)	74 ± 9	EVT	2.9	≤ 98 *vs*. >98	Death, CLTI, amputation, CVD readmission	MACLEs 2.24 (1.19–4.24)#	Age, fat-free mass index, hyperlipidemia, previous CAD, CLI, eGFRcys, hsCRP
Mii et al. (14)	Japan	R	IC 188 (75)	69–79	Bypass surgery	4.0	Per 10 units decrease	—	Total death 1.43 (0.92–2.17)	Age, ABI, DM, CAD, COPD, late time period
Mii et al. (12)	Japan	R	CLTI 373 (67.3)	73.8 ± 8.9	Bypass surgery	2.7	<92 *vs*. ≥92	—	Total death: 2.26 (1.50–3.41)	Age, ABI, end-stage renal disease, non-ambulatory
Jhang et al. (13)	Taiwan	P	PAD 232 (47)	85 ± 4.2	EVT	2.66	<90.3 *vs*. ≥90.3	—	Total death 3.07 (1.45–6.52)	Ambulatory status, congestive heart failure, CVA, chronic limb-threatening ischemia, dialysis, NLR, TC
Matsuo et al. (21)	Japan	P	PAD 1219 (75.7)	73 (67–79)	EVT, bypass surgery, medications	6.1	Per 10 units decrease	—	Total death 1.48 (1.34–1.63) MACLEs 1.34 (1.10–1.48)	Age, sex, ABI, CLI, stroke or TIA, eGFR, C-reactive protein, d-dimer, statin, aspirin, revascularization
Yamaguchi et al. (22)	Japan	R	PAD 2246 (71.7)	73.2 ± 9.3	EVT	2.0	Per 10 units decrease	Death, stroke, MI, amputation, limb surgery, EVT, CLTI admission	MACLEs 1.37 (1.25–1.50)#	Comorbidities, procedural parameters, and type of drug usage
Shiraki et al. (15)	Japan	P	CLTI 499 (67.7)	73 ± 10	EVT, bypass surgery	3.0	Per 10 units decrease	—	Total death 1.26 (1.02–1.56) Amputation 0.96 (0.65–1.43)	Age, sex, non-ambulatory status, smoking, DM, regular dialysis, heart failure, tissue loss, BMI, TC, lymphocyte count, albumin
Li et al. (16)	USA	R	CLTI 255 (61.8)	71 (61–81)	EVT	1.2	≤ 94 *vs*. >94; Per 10 units decrease	Death, amputation, TVR	Total death 2.17 (0.98–5.00) 1.48 (1.10–2.59) MACLEs 2.27 (1.42–3.70) 1.34 (1.10–1.79) Amputation 1.41 (0.52–3.85) 1.34 (0.81–2.37)	WBC, hemoglobin, CKD, DM, WIFI clinical stage, pre-intervention ABI

*HR, hazard ratio; CI, confidence intervals; SD, standard deviation; R, retrospective; P, prospective; PAD, peripheral artery disease; EVT, endovascular therapy; GNRI, geriatric Nutritional Risk Index; MACLEs, major adverse cardiovascular and leg events; ABI, ankle-brachial index; TVR, target vessel revascularization; hsCRP, high-sensitivity C-reactive protein; Cys, cystatin C; CLTI, chronic limb threatening ischemia; IC, intermittent claudication; CVA, cerebrovascular accident; TIA, transient ischemic attack; NLR, neutrophil-lymphocyte ratio; MI, myocardial infarction; CAD, coronary artery disease; COPD, chronic obstructive pulmonary disease; CKD, chronic kidney disease; WBC, white blood cell; WIFI, wound, ischemia, and foot infection; TC, total cholesterol; CVD, cardiovascular disease; BMI, body mass index; DM, diabetes mellitus; eGFR, estimated glomerular filtration rate; # Results pooled from subgroups using a fixed-effect model*.

**Table 2:** Methodological quality of the included studies.

**Author/year**	**Representativeness of the exposed cohort**	**Selection of the non-exposed cohort**	**Ascertainment of exposure**	**Demonstration that outcome was not present at study start**	**Comparability of cohorts based on the design or analysis**	**Assessment of outcome**	**Enough follow-up periods (≥3 years)**	**Adequacy of follow-up of cohorts**	**Overall NOS scores**
Luo et al. (9)		⋆	⋆	⋆	⋆⋆	⋆	⋆		7
Yokoyama et al. (11)	⋆	⋆	⋆	⋆	⋆⋆	⋆		⋆	8
Mii et al. (14)		⋆	⋆	⋆	⋆⋆	⋆	⋆	⋆	8
Mii et al. (12)		⋆	⋆	⋆	⋆⋆	⋆		⋆	7
Jhang et al. (13)	⋆	⋆	⋆	⋆	⋆	⋆		⋆	7
Matsuo et al. (21)	⋆	⋆	⋆	⋆	⋆	⋆	⋆	⋆	8
Yamaguchi et al. (22)	⋆	⋆	⋆	⋆	⋆	⋆		⋆	7
Shiraki et al. (15)		⋆	⋆	⋆	⋆⋆	⋆	⋆	⋆	8
Li et al. (16)		⋆	⋆	⋆	⋆⋆	⋆		⋆	7

*NOS, Newcastle-Ottawa Scale*.

### Categorical Analysis of GNRI Score

**Figure 2:**
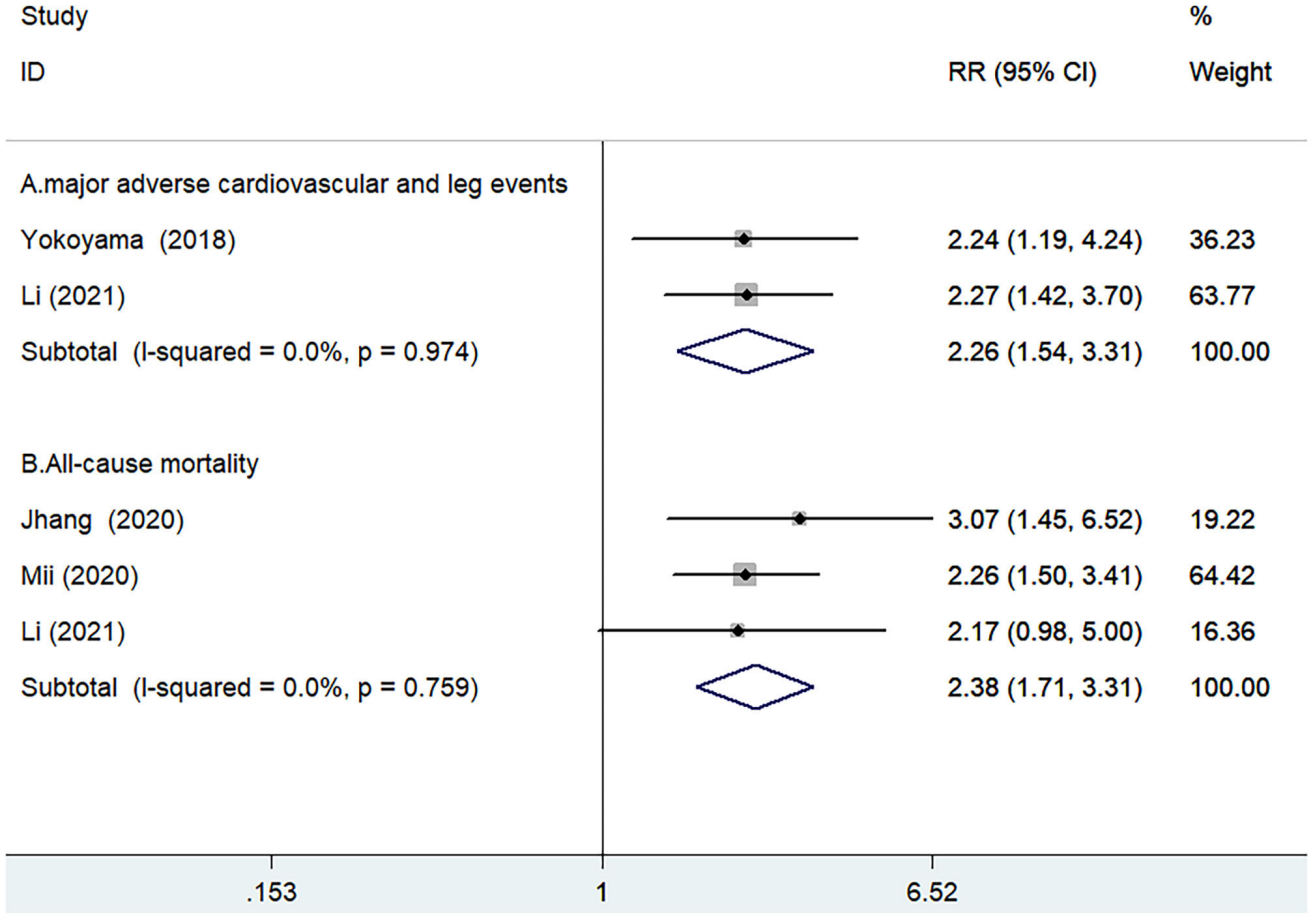
Forest plots showing pooled risk ratio (RR) with 95% CI of MACLEs **(A)** and all-cause mortality **(B)** for the low *vs*. high GNRI score.

Two studies (11, 16) reported the value of GNRI in predicting MACLEs by categorical analysis. A fixed-effect model meta-analysis indicated that the pooled adjusted RR of MACLEs was 2.26 (95% CI 1.54–3.31) for the low *vs*. high GNRI score, without evidence of significant heterogeneity (I^2^ = 0.0%; *p* = 0.971; Figure 2A).

Three studies (12, 13, 16) reported the value of GNRI in predicting all-cause mortality by categorical analysis. A fixed-effect model meta-analysis indicated that the pooled adjusted RR of all-cause mortality was 2.38 (95% CI 1.71–3.31) for the low *vs*. high GNRI score, without evidence of significant heterogeneity (I^2^ = 0.0%; *p* = 0.759; Figure 2B). Leave-out one-study sensitivity analysis did not alter the originally statistical significance of the pooling risk estimate.

### Continuous Analysis of GNRI Score

**Figure 3:**
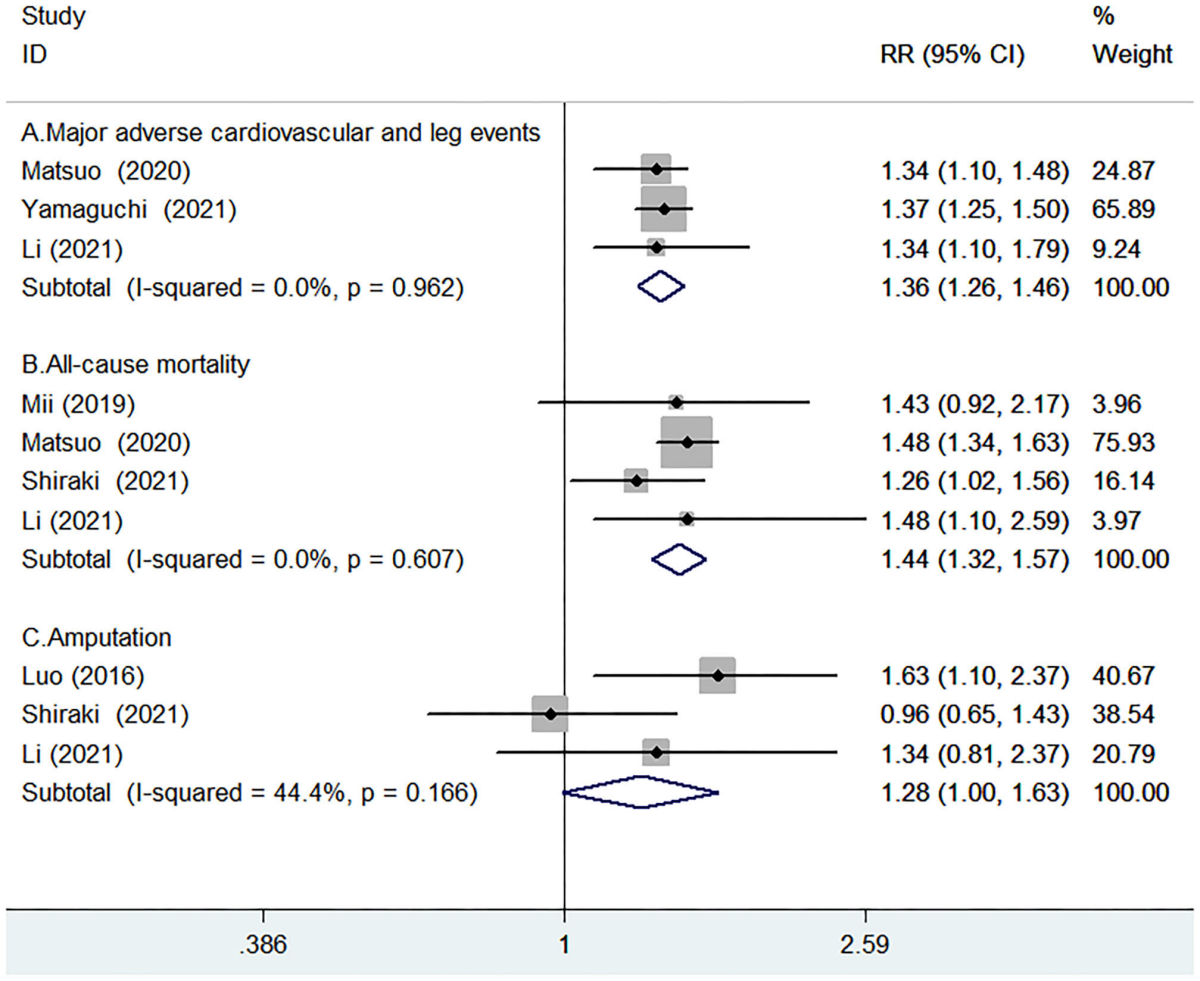
Forest plots showing pooled RR with 95% CI of major adverse cardiovascular and leg events (MACLEs) **(A)**, all-cause mortality **(B**), and amputation **(C)** for per-10-units Geriatric Nutritional Risk Index (GNRI) score decrease.

Three studies (16, 21, 22) reported the value of GNRI in predicting MACLEs by continuous analysis. A fixed-effect model meta-analysis indicated that per 10 units GNRI scores decrease was associated with a higher risk of MACLEs (RR 1.36; 95% CI 1.26–1.46; I^2^ = 0.0%; *p* = 0.962; Figure 3A). The originally statistical significance of the pooling risk estimate was stable in the leave-out one-study sensitivity analysis.

Four studies (14–16, 21) reported the value of GNRI in predicting all-cause mortality by continuous analysis. A fixed-effect model meta-analysis indicated that the per-10-units GNRI scores decrease was associated with an increased risk of all-cause mortality (RR 1.44; 95% CI 1.32–1.57; I^2^ = 0.0%; *p* = 0.607; Figure 3B). Leave-out one study sensitivity analysis demonstrated the robustness of the pooling risk estimate.

Three studies (9, 15, 16) reported the value of GNRI in predicting amputation by continuous analysis. As shown in Figure 3C, there was no significant heterogeneity between studies (I^2^ =44.4%; *p* = 0.166). A fixed-effect model meta-analysis showed that the per-10-units GNRI score decrease was not significantly associated with a higher risk of amputation (RR 1.28; 95% CI 1–1.63; *p* = 0.051). In the sensitivity analysis, the pooled RR of amputation ranged from 1.06 to 1.26 and the low 95% CI ranged from 0.79 to 1.12, suggested that the pooling risk estimate was potentially unreliable.

### Publication Bias

Due to less than recommended arbitrary number of 10 studies, we did not run the Begg's test and Egger's test to check the likelihood of publication bias (23).

## Discussion

This is the first meta-analysis to assess the predictive value of the GNRI score in patients with lower extremity PAD. The principal findings of the current meta-analysis consolidated the accumulating evidence that low GNRI independently predicted MACLEs and all-cause mortality in patients with lower extremity PAD. Patients with lower extremity PAD with low GNRI scores conferred a 2.26-fold and 2.38-fold increased risk of MACLEs and all-cause mortality, respectively. Moreover, per 10 units GNRI score decrease was associated with 36% and 44% higher risk of MACLEs and all-cause mortality, respectively.

However, the value of the GNRI score by continuous analysis in predicting amputation was not statistically significant. Moreover, malnutrition defined by the GNRI <92 was significantly associated with cerebrovascular or cardiovascular mortality in CLTI patients after revascularization (24). These findings suggest that nutritional status determined by the GNRI may provide some important prognostic information in patients with lower extremity PAD.

Several nutritional scoring systems, including the GNRI, Controlling Nutritional Status (CONUT) (11, 13, 25), and prognostic nutritional index (PNI) (26), have been applied to assess the nutritional status in patients with lower extremity PAD. However, there is no census on which nutritional tool has the best predictive value in patients with PAD. Yokoyama et al.' s study (11) indicated that the risk estimate for the CONUT-based moderate to severe malnutrition was higher than the GNRI-based in predicting MACLEs in patients with PAD undergoing endovascular therapy. By contrast, Jhang et al.' s study (13) showed that the value for the GNRI-based moderate to severe malnutrition was stronger than the CONUT-based in predicting 2-year mortality in patients with lower extremity arterial disease. Future well-designed studies are warranted to directly compare which nutritional scoring-based malnutrition has the best predictive value in patients with lower extremity PAD.

The exact mechanisms underlying the predictive value of malnutrition defined by the GNRI score have not been well-characteristic in patients with lower extremity PAD. Albumin and body mass index (BMI) are crucial components of the GNRI formula. Low albumin level was associated with postoperative death and prolonged length of hospital stay after lower extremity procedures for lower extremity PAD (27, 28). Patients with underweight BMI with PAD had worse in-hospital mortality and more adverse outcomes after endovascular therapy (29). The GNRI score represents the combined albumin and BMI, which can synergetically improve the predictive value.

Considering the high prevalence and impact of malnutrition on the adverse prognosis, patients with lower extremity PAD should be routinely assessed for the nutritional status. Our meta-analysis highlights the importance to determine the nutritional status using the GNRI score in patients with lower extremity PAD. Patients with lower extremity PAD with malnutrition estimated by the low GNRI score should be considered a high-risk group and received nutrition-based treatment strategies. However, there are no well-designed clinical trials to support the benefits of nutrition-based treatment strategies in this group of patients. Future clinical trials are required to examine whether nutritional intervention can improve prognosis in patients with malnutrition with lower extremity PAD.

A few potential limitations should be mentioned in this meta-analysis. First, the number of included studies in the analyzed outcomes was relatively small, which prevents us to conduct subgroup analysis according to the severity of lower extremity PAD, treatment strategies, or degree of malnutrition defined by GNRI score. Second, the cutoff values for the categorical analysis of GNRI score varied across the analyzed studies. Therefore, the optimal threshold of a low GNRI score required further determination. Third, the definition of MACLEs was not identical in the included studies, which could have potentially affected the pooling risk estimate. Finally, we failed to perform subgroup analysis according to the intermittent claudication or CLTI and degree of malnutrition defined by GNRI score due to insufficient data.

## Conclusion

Low GNRI may be an independent predictor of adverse outcomes in patients with lower extremity PAD. Routine screening of nutritional status using the GNRI may provide important prognostic information in patients with lower extremity PAD.

## Data Availability Statement

The original contributions presented in the study are included in the article/supplementary material, further inquiries can be directed to the corresponding authors.

## Author Contributions

YF and XW: study conception/design and interpretation of data. GL and CZ: literature search, data extraction, and quality assessment. YJ and PW: statistical analysis. GL: writing the manuscript. All authors approved the version of the manuscript.

## Funding

This work was supported by Suqian Sci & Tech Program (K202014), Jiangsu Six High Peak Talent Fund (WSW236), and Zhenjiang Key Research and Development Fund (SH2021038).

## Conflict of Interest

The authors declare that the research was conducted in the absence of any commercial or financial relationships that could be construed as a potential conflict of interest.

## Publisher's Note

All claims expressed in this article are solely those of the authors and do not necessarily represent those of their affiliated organizations, or those of the publisher, the editors and the reviewers. Any product that may be evaluated in this article, or claim that may be made by its manufacturer, is not guaranteed or endorsed by the publisher.
